# Microhydration of phenylboronic acid and its hydration free energy

**DOI:** 10.1007/s00894-025-06436-2

**Published:** 2025-07-19

**Authors:** Doungous Sale, Alhadji Malloum, Mama Nsangou, Jean Jules Fifen, Jeanet Conradie

**Affiliations:** 1 Department of Physics, Faculty of Science, University of Maroua, PO BOX 46, Maroua, Cameroon; 2 Department of Chemistry, University of the Free State, PO BOX 339, 9300 Bloemfontein, South Africa; 3 Department of Physics, Faculty of Science, The University of Ngaoundere, PO BOX 454, Ngaoundere, Cameroon; 4 Department of Chemistry, UiT - The Arctic University of Norway, N-9037 Tromsø, Norway

**Keywords:** Free energy of solvation, Solvation of phenylboronic acid, Hydrogen bonding network, QTAIM analysis, Temperature-effects

## Abstract

****Context**:**

Understanding the interactions of phenylboronic acid with its surrounding water molecules is essential for several applications in solvated systems. In the present work, we investigated the microhydration of the phenylboronic acid (PBA) and calculated its hydration free energy using the cluster continuum solvation model. Microhydration of PBA has not been investigated previously in the literature. It requires the structures of PBA to be surrounded by *n* explicit water molecules (PBA-W$_n$). The results show that the B(OH)$_2$ unit of phenylboronic acid forms clusters with water molecules that are similar to those of neutral water clusters. The QTAIM analysis shows that the structures of phenylboronic acid-water clusters are stabilized by strong OH$\cdots$O and weak CH$\cdots$O hydrogen bonds. In addition to QTAIM analysis, NBO analysis was also performed on the most stable configurations to better understand the delocalization of electron density from donor to proper acceptor within the compound. In addition, we found that the most stable structures dominate the population of the clusters for temperatures from 20 to 400 K. Finally, using the structures of the microhydrated phenylboronic acid, we estimated the free energy of hydration and the enthalpy of hydration of PBA. At room temperature, the phenylboronic acid’s free energy and enthalpy of hydration are respectively evaluated to be $-$72.1 kcal/mol and $-$85.5k cal/mol. Assessment of temperature effects on the free energy and the enthalpy of hydration shows that the enthalpy is temperature-independent, while the free energy increases linearly with temperature.

****Methods**:**

Initial configurations of PBA-W$_n$ have been generated using classical molecular dynamics and subsequently optimized using the level of theory, $\omega$B97X-D/def2-TZVP. Optimizations, frequency calculations, and NBO analysis are performed using the Gaussian 16 suite of programs. On the most stable configurations, we have performed the quantum theory of atoms in molecules (QTAIM) analysis to get insights into the hydrogen bond network of PBA-W$_n$. QTAIM is performed using AIMAll software. Thermodynamic properties as a function of temperature are evaluated using a homemade FORTRAN code-named **TEMPO**.

## Introduction

Phenylboronic acid is defined as an organic aromatic compound with two hydroxyl groups, soluble in most polar organic solvents. Phenylboronic acid (PBA) is soluble in water with a dissociation constant of 8.8 [1]. The dissociation of PBA in water is influenced by pH. PBA tends to form boronate anions at higher pH levels, which are more soluble [1, 2]. It is a valuable compound used in industries for multiple needs [3, 4]. We use, for example, phenylboronic acid and its derivatives in organic synthesis and several pharmaceutical drugs for the cancer and diabetic treatment of many patients worldwide. Phenylboronic acid derivatives have been investigated for their potential medicinal properties, particularly in developing anti-cancer and anti-inflammatory drugs. Many authors reported that phenylboronic acid is essential in diabetes treatment. Huang et al. [5] reported that the injection of phenylboronic acid into the human body automatically releases the corresponding amount of insulin according to the blood glucose level in the human body. Phenylboronic acid can bind to sugars and other molecules through boronate ester formation. This property is used in various sensors and assays to detect glucose and other analytes. Kurnia et al. [6] also reported that Boronic acid is studied for the molecular recognition and sensing of various saccharides, polysaccharides, glycoproteins, and nucleosides. In particular, saccharides and polysaccharides are commonly detected in disease diagnostics, such as diabetes. The applications of phenylboronic acid mentioned above occur in the human body, in aqueous solution. Therefore, understanding the interaction of phenylboronic acid with the surrounding water molecules becomes essential. In this work, we studied phenylboronic acid from different perspectives, namely, the microhydration of phenylboronic-water clusters (PBAWn) and the free energy of solvation of PBA.

Exploration of the literature shows that there is no reported study on the microhydration of the phenylboronic acid (PhB(OH)$_{2}$(H$_{2}$O)$_n$ or simply PBAW$_n$). However, studies on the microhydration of similar molecular systems have been reported previously. Malloum and Conradie [7] reported the microhydration of phenol (PhOH(H$_{2}$O)$_n$) at the $\omega$B97X-D/aug-cc-pVDZ level of theory. The authors have investigated the hydrogen bond networks of PhOH(H$_{2}$O)$_n$ for *n* varying from 1 to 12 explicit water molecules [7]. In addition to phenol, microhydration of aminobenzoic acid has been investigated by several authors using different computational levels of theory [8–10]. Anni and coworkers [10] found that the structure of the hydrated aminobenzoic acid is highly stable when the carboxyl group interacts with the molecules of water. Besides, it has been found that the configurations are stabilized by strong hydrogen bonds, OH$\cdots$O and weak hydrogen bonds, CH$\cdots$O and OH$\cdots \pi$ hydrogen bonds [10]. Microhydration of several other amino acids has been reported in the literature. Microhydration of glycine [11, 12], and alanine [13] has been reported by several authors. Several other studies are reported in the literature on the microhydration of biomolecules and molecules for interstellar interests. These studies highlight the importance of understanding the interaction of the studied molecules with their surrounding molecules (water molecules). The lack of such a study of the phenylboronic acid justifies the need to perform the current investigation.

The free energy of solvation and enthalpy of solvation of phenylboronic acid in water molecules have not been reported previously. Nevertheless, the free energy of solvation of similar systems has been reported by several authors using different methodologies. Gallicchio and coworkers reported the hydration free energy of several molecules [14] based on a surface generalized Born continuum dielectric electrostatic model. Ranaudo et al. [15] determined the phenylboronic acid free energy of hydration from the anionic form PhB(OH)$_{3}^{-1}$ using three different models. The evaluated hydrated free energy with these three different models is reported to be $-$82.7 kcal/mol, $-$67.2 kcal/mol, and $-$67.6 kcal/mol, respectively [15]. The reported energies are evaluated at room temperature.

The literature observation shows that the geometries and the bonding interaction networks of PBAW*n* have been limited to the case of the substituted phenylboronic acid clusters. Moreover, for the PBAW*n* clusters, there is no evidence reporting on the exploration of global minimum potential energy surfaces (PESs). It is important to note that the accuracy of any investigation on the solvation of phenylboronic acid-water clusters depends on the accuracy of all the geometries that may be identified. Therefore, it becomes essential to carry out a complete study of the clusters of PBA(H$_2$O)$_n$. Hence, the PESs of the PBAW*n* clusters, for $n =1-10$, are explored. We optimized the retained configurations using a dispersion-corrected DFT functional, $\omega$B97X-D, combined with the basis set, def2-TZVP. In addition, we have performed the quantum theory of atoms in molecule analysis on the most stable conformers of the clusters of phenylboronic acid in water to identify the interactions between phenylboronic acid and solvent molecules(water). NBO analysis was also performed on the most stable configurations to understand better the electron density delocalization from the donors to the acceptors. The absolute energies of hydration and the thermodynamic properties are reported for temperatures varying from 20 to 400 K.

## Methodology

This section begins by presenting the methodology used in this work to sample initial configurations (see “Samplingwith ABCluster” section). To compute the enthalpy of solvation and the free energy of solvation of phenylboronic acid, we used in this work the solvation model of density(SMD), presented as a cluster continuum model in the “Calculationof the hydration enthalpy and free energy” section. In the “Computational details” section are presented the computational details, which present the choice of the software used, the computational level of theory, and details to ameliorate the accuracy of the calculations.

### Sampling with ABCluster

To achieve this work’s objectives, we identify the geometries of the hydrated phenylboronic acid clusters, PBAWn, $n = 1-10$, with low-lying energy. Hence, we started by generating the different possible geometries of clusters. Initial conformers for the different cluster sizes are sampling using the ABCluster code of Zhang and Dolg [16, 17]. ABCluster generates all possible conformers and ranges them from the most stable to the least stable conformers based on classical energy. The classical energy used by ABCluster is constituted of Lennard–Jones and electrostatic potential. The different parameters are based on the CHARMM’s force field [18]. We can find in the previous works reported by Malloum et al. [19, 20] more details on how the configurations are sampled. ABCluster has been successfully applied to study neutral molecular clusters, including acetonitrile clusters [21], neutral water clusters [22], thiophene clusters [23, 24], and dimethylsulfoxide (DMSO) clusters [25]. In addition, the reader is advised to read the original papers of Zhang and Dolg [16, 17] for more details on ABCluster.

### Calculation of the hydration enthalpy and free energy

The determination of the free energy of solvation and the enthalpy of solvation has been performed at the cluster’s solvation model of density (SMD). In this approach, SMD, the phenylboronic acid is explicitly surrounded by a few molecules of water, while a dielectric continuum medium represents the remaining solvent. The phenylboronic acid solvation can be defined by Eq. 1.

1 $$\begin{aligned} \text {PBA}(g)+(\text {H}_{2}\text {O})_{n}(s) \rightarrow \text {PBA}(\text {H}_{2}\text {O})_{n}(s) \end{aligned}$$

when PBA is the phenylboronic acid.

Then, we determine the absolute free energy of hydration and the absolute enthalpy of hydration of the phenylboronic acid using Eqs. 2 and 3, respectively.

2 $$\begin{aligned} \varDelta \text {G}_{s}(\text {PBA})_{n}= \varDelta \text {G}_{s}[\text {PBA}(\text {H}_{2}\text {O})_{n}]-\varDelta \text {G}_{s}[(\text {H}_{2}\text {O})_{n}]-\varDelta \text {G}_{g}[\text {PBA}], \end{aligned}$$

3 $$\begin{aligned} \varDelta \text {H}_{s}(\text {PBA})_{n}= \varDelta \text {H}_{s}[\text {PBA}(\text {H}_{2}\text {O})_{n}]-\varDelta \text {H}_{s}[(\text {H}_{2}\text {O})_{n}]-\varDelta \text {H}_{g}[\text {PBA}], \end{aligned}$$

Here, the different subscripts *g* and *s* represent the gas phase and the implicit water solvent, respectively. Thus, the free energy of hydration and the enthalpy of hydration of phenylboronic acid in water is determined when the evaluated values of $\varDelta \text {G}_{s}(\text {PBA})_{n}$ and $\varDelta \text {H}_{s}(\text {PBA})_{n}$ are constant when the cluster size n increases. It is important to note that $\varDelta \text {G}_{s}[\text {PBA}(\text {H}_{2}\text {O})_{n}]$ and $\varDelta \text {G}_{s}[(\text {H}_{2}\text {O})_{n}]$ are calculated using the weighted Boltzmann distribution of all possible clusters of the corresponding size *n*. This also applies to the enthalpies. The Boltzmann weighted free energy is calculated using Eq. 4.

4 $$\begin{aligned} \varDelta \text {G(T)}= &   \sum \limits _{i} \varDelta \text {G}_{i}(\text {T})\text {P}_{i}(\text {T}) \end{aligned}$$

5 $$\begin{aligned} \text {P}_{i}(\text {T})= &   \dfrac{\exp (-\beta \varDelta \text {G}_{i}(\text {T}))}{\sum \limits _{i}\exp (-\beta \varDelta \text {G}_{i}(\text {T}))}, \end{aligned}$$

Here, $\beta$ is given by the relation $\beta =1/k_BT$, and $k_B$ represents the constant of Boltzmann. $\Delta G_i$ represent the free energy of the $i-th$ configuration of the corresponding size *n* of the clusters. It is important to note that the Boltzmann distribution is only valid when a complete set of structures is known. However, based on our experience, the structures that significantly contribute to the distribution of the clusters are within 2.0 kcal/mol. In this work, we have explored all the structures within 2.0 kcal/mol from the most stable to ensure the validity of the Boltzmann distribution.

From the Eqs. 2 and 3, it comes out that the hydration free energy and enthalpy of PBA are evaluated from the evaluation of the different geometries of the hydrated PBA clusters, PBA(H$_{2}$O)$_n$ and the conformers of the neutral water clusters. The different geometries clusters of the hydrated phenylboronic acid are sampled and optimized in this work, and the different conformers of the clusters of neutral water optimized in this work are also reported by Malloum et al. [22, 26]. Although not explicitly shown, Eqs. 2 and 3 are dependent on temperature. The free energy and enthalpy of PBA(H$_{2}$O)$_n$ and (H$_{2}$O)$_n$ are evaluated using the free energies of the different conformers, $\varDelta \text {G}_{i}(\text {T})$, weighted by their relatives population, P$_{i}$(T) (see Eq. 5). Thus, for a given cluster, the participation of the conformers in the final free energy is conditioned by its Boltzmann weight P$_{i}$(T). In the “Rela-tive population of the hydrated phenylboronic acid” section, we will see that most of the isomers do not participate in the evaluated relative population, or their relative population contributions are negligible. It is important to indicate that free energies $\varDelta \text {G}_{i}(\text {T})$ of differently located conformers and the Boltzmann weight P$_{i}$(T) are evaluated using the Fifen and coworkers Tempo code [27, 28].

**Fig. 1:**
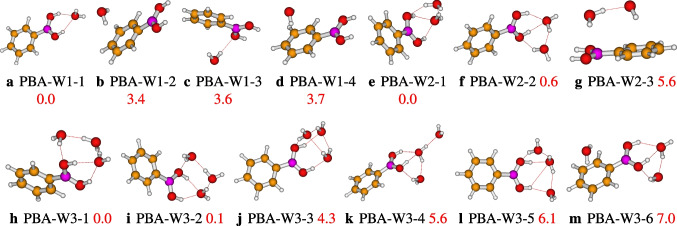
Different conformers of the phenylboronic acid in water for *n* = 1, 2, and 3, optimized at the $\omega$B97X-D/def2-TZVP level of theory. The relative zero-point corrected energy of isomers in kcal/mol is represented by the numbers in red color

### Computational details

We used ABCluster to sample the different conformers of the solvated phenylboronic acid clusters, and the sampled conformers have been completely optimized using the level of theory, $\omega$B97X-D/def2-TZVP [29, 30]. To be sure that the geometries located are the most stable, the calculation of the frequencies has also been performed at the same level of theory, and the results show that no imaginary frequency is found for the located geometries. However, these frequencies are also involved in the calculation of the clusters of solvated phenylboronic acid properties: free energies and enthalpies. Gaussian 16 program suite [31] is used in the optimizations and frequency calculation. The *tight* optimization option is used for the accurate optimizations, and in the case of the accurate integrals, we used *ultrafine* grid. The implicit solvation is modeled using the implicit solvation model based on the density (SMD). Quantum theory of atoms in molecules (QTAIM) analysis is performed on the most stable configurations using the AIMAll program [32]. Relative population (or stability) of the clusters as a function of temperature, as well as the free energy and enthalpy as a function of temperature, are calculated using our home-made FORTRAN code-named **TEMPO** [27, 28]. The program’s output has been compared and validated against Gaussian calculated values [27].

## Results and discussions

This section presents the different optimized structures of the phenylboronic acid-water clusters in the “Structures ofthe hydrated phenylboronic acid” section. The optimization of these configurations has been performed using the level of theory, $\omega$B97X-D/def2-TZVP, and the retained configurations are classified in function of their gas phase relative electronic energies, including the corrections of zero-point energy (ZPE). In the “QTAIM analysis of non-covalentbondings” section, we examine the non-covalent bondings stabilizing on the investigated clusters using QTAIM analysis, after presenting the stability in the “Structures of thehydrated phenylboronic acid” section. In the “Relative pop-ulation of the hydrated phenylboronic acid” section, we present the relative population of the configurations that contribute significantly to the population of the clusters. The configurations of neutral water clusters are presented by order of stability according to their relative energies in the “Conformers of neutral water clusters” section. Finally, for different temperatures varying from 20 to 400 K, we present the evaluated free energy of hydration and enthalpy of hydration of the phenylboronic acid in the “Hydration enthalpy andfree energy” section.

### Structures of the hydrated phenylboronic acid

It is reported in Fig. 1 the optimized geometries of the hydrated phenylboronic acid monomer (PBAW1), dimer, and trimer. We have four configurations of the solvated phenylboronic acid monomer, which are stable. The most stable configuration is PBA-W1-1, and the least stable is PBA-W1-4, with relative energy varying from 0.0 to 3.7 kcal/mol, we can see that in Fig. 1. In PBA-W1-1, the interaction between the solvent molecule(water) and the phenylboronic acid molecule presents two strong OH$$\cdots $$O hydrogen bonds. In addition, the B(OH)$$_2$$ group of phenylboronic acid acts as a hydrogen bond donor, while the water molecule is the hydrogen bond acceptor (see Fig. 1). The isomer PBA-W1-1 belongs to the $$C_{2v}$$ symmetry point group. For PBA-W1-4, the solvent molecule(water) and the phenylboronic acid molecule interact with only one strong OH$$\cdots \pi $$ hydrogen bond. As far as the PBAW2 is concerned, we have initially optimized ten geometries, among which three geometries have been identified as different from each other with energy varying from 0.0 to 5.6 kcal/mol (see Fig. 1). The PBA-W2-1 is the most stable geometry of the hydrated phenylboronic acid dimer among those retained. In PBA-W2-1, the interaction between the solvent molecules (water) and the phenylboronic acid molecule presents more than two strong OH$$\cdots $$O hydrogen bonds. In addition, we can see the two solvent molecules interact with a strong hydrogen bonding, OH$$\cdots $$O, and act like a hydrogen bond acceptor-donor (see Fig. 1). Initially, we have optimized ten geometries of the solvated phenylboronic acid trimer (PBAW3). After optimization, we have identified six stable geometries which are different from one another, and in Fig. 1 are reported the six located geometries with energy varying from 0.0 to 7.0 kcal/mol. The PBA-W3-1 is the trimer’s most stable isomer (see Fig. 1). For this isomer, a double cyclic bondings hydrogen OH$$\cdots $$O network is formed between the three solvent molecules and the B(OH)$$_{2} $$ group. The three water molecules also act as hydrogen bond acceptor-donor (see Fig. 1).

**Fig. 2:**
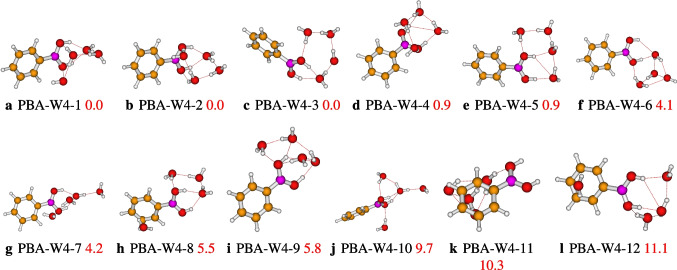
Different conformers of the phenylboronic acid in water for *n* = 4, as optimized at the $\omega$B97X-D/def2-TZVP level of theory. The relative zero-point corrected energy of isomers in kcal/mol is represented by the numbers in red color

Regarding the hydrated phenylboronic acid tetramer, twelve conformers are the most stable. The located conformers show that the relative energies vary from 0.0 to 11.1 kcal/mol that can be seen in Fig. 2. PBA-W4-1, PBA-W4-2, and PBA-W4-3 are iso-energetically the most stable structures. These most stable structures show the strong OH$\cdots$O hydrogen bonds characteristic of high stability. Besides, PBA-W4-1 presents two weak bondings hydrogen represented by CH$\cdots$O (see Fig. 2). There is also the presence of the OH$\cdots \pi$ interaction (see Fig. 9). So, the fact that PBA-W4-1, PBA-W4-2, and PBA-W4-3 present extra bond interactions additional to the cyclic hydrogen bonds, OH$\cdots$ O is one of the criteria indicating the most stability of the structure. In those isomers, PBA-W4-1, PBA-W4-2, and PBA-W4-3, the interactions between the four solvent molecules and the phenylboronic acid B(OH)$_{2}$ group acted like proton donor-acceptor.

**Fig. 3:**
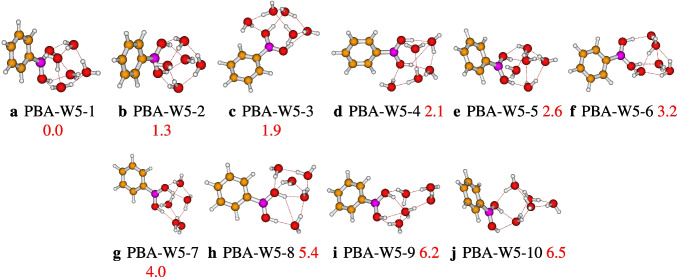
Different conformers of the phenylboronic acid in water for *n* = 5, optimized at the $\omega$B97X-D/def2-TZVP level of theory. The relative zero-point corrected energy of isomers in kcal/mol is represented by the numbers in red color

Initially, we optimized ten geometries of the phenylboronic acid in water pentamer using the level of theory, $\omega$B97X-D/def2-TZVP. After optimization, the ten geometries are stable and present different geometric shapes. The located conformers are classified from the most stable geometry to the least, with the relative energies varying from 0.0 to 6.5 kcal/mol, which are reported in Fig. 3. The PBA-W5-1 isomer is found to be the most stable structure and has the highest number of strong OH$\cdots$O hydrogen bonds. Besides, its QTAIM analysis shows the presence of the O$\cdots$C bond interaction (see Fig. 9). For all the configurations, it can be seen that the interaction between the five water molecules and the group B(OH)$_{2}$ acts as a proton donor-acceptor. Besides the strong bondings hydrogen OH$\cdots$O, the isomers PBA-W5-3 and PBA-W5-4 present a weak bonding hydrogen CH$\cdots$O.

**Fig. 4:**
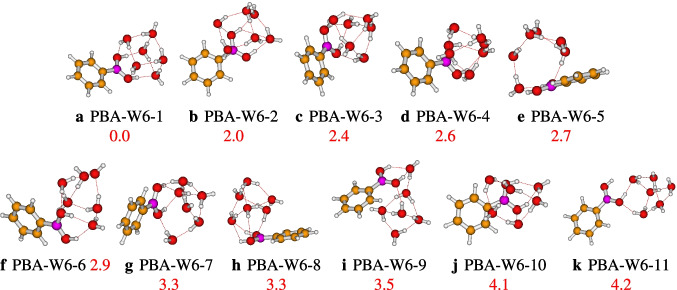
Different conformers of the phenylboronic acid in water for *n* = 6, optimized at the $\omega$B97X-D/def2-TZVP level of theory. The relative zero-point corrected energy of isomers in kcal/mol is represented by the numbers in red color

After optimization, eleven geometries for PBA-W$_6$ are retained to be stable, which are different. The retained structures’ relative energies vary from 0.0 to 4.2 kcal/mol. The retained geometries are reported in Fig. 4, and PBA-W6-1 is the most stable geometry, which is stabilized only by strong hydrogen bonds, OH$\cdots$O. The B(OH)$_{2}$ group and the six water molecules generate a configuration similar to a neutral water octamer. The two OH groups in B(OH)$_{2}$ are acting like separate hydroxyl groups. The generated structure of PBA-W6-1 has a cubic-like configuration similar to the water octamer [33, 34]. For all of the reported configurations, the interaction between the molecules of solvent and the B(OH)$_{2}$ group occurs through the proton donor-acceptor. Besides the strong bondings hydrogen OH$\cdots$O, the isomers PBA-W6-5, PBA-W6-8, PBA-W6-9, and PBA-W6-10 have a weak OH$\cdots \pi$ hydrogen bonds (see Fig. 4). The less stable isomer is PBA-W6-11, which has the least number of OH$\cdots$O hydrogen bonds.

**Fig. 5:**
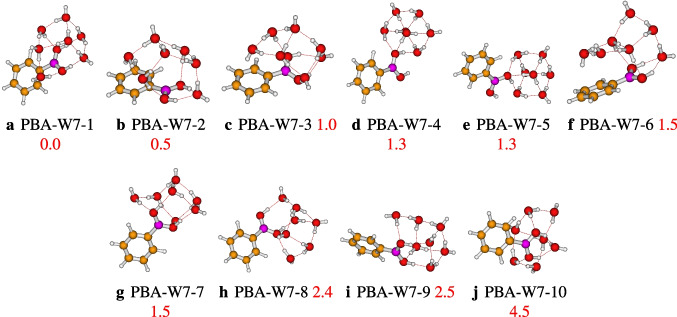
Different conformers of the phenylboronic acid in water for *n* = 7, optimized at the $\omega$B97X-D/def2-TZVP level of theory. The relative zero-point corrected energy of isomers in kcal/mol is represented by the numbers in red color

Optimization of geometries generated by ABCluster led to ten isomers of the phenylboronic acid in water heptamer. The relative energies of the located geometries vary from 0.0 to 4.5 kcal/mol, and the different geometries retained are reported in Fig. 5. PBA-W7-1 is the most stable isomer, with strong hydrogen bonds OH$\cdots$O and two weak hydrogen bonds CH$\cdots$O. According to the OH$\cdots$O hydrogen bond network, a similarity between the interactions in PBA-W7-1 and neutral water nonamer is observed [22, 35, 36]. In addition to the strong bondings hydrogen $\cdots$O and weak bondings hydrogen CH$\cdots$O, the QTAIM analysis of PBA-W7-1 shows the presence of OH$\cdots \pi$ hydrogen bonds (see Fig. 9). For all the retained geometries, the results show that the two groups (the solvent molecules and B(OH)$_{2}$) interact as proton donor-acceptors.

**Fig. 6:**
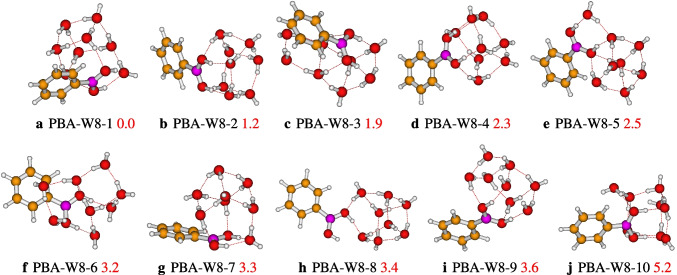
Different conformers of the phenylboronic acid in water for *n* = 8, optimized at the $\omega$B97X-D/def2-TZVP level of theory. The relative zero-point corrected energy of isomers in kcal/mol is represented by the numbers in red color

**Fig. 7:**
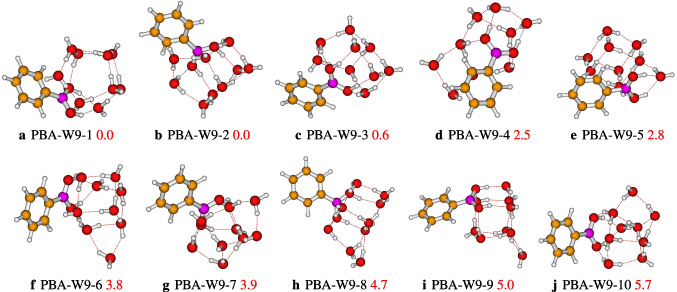
Different conformers of the phenylboronic acid in water for *n* = 9, optimized at the $\omega$B97X-D/def2-TZVP level of theory. The relative zero-point corrected energy of isomers in kcal/mol is represented by the numbers in red color

After optimization of initial geometries, ten structures out of eleven are found to be stable according to their relative energies, and the structures are different. The relative energies of the retained structures vary from 0.0 to 5.2 kcal/mol, which are reported in Fig. 6. Between the retained structures, the most stable geometry is PBA-W8-1, which has a cubic type configuration, with strong bondings hydrogen, OH$\cdots$O, and weak bondings hydrogen, CH$\cdots$O. Furthermore, the QTAIM analysis of PBA-W8-1 shows the presence of another bond, which is O$\cdots$C (see Fig. 9). The majority of the reported configurations present weak CH$\cdots$O hydrogen bonds except for three isomers (PBA-W8-2, PBA-W8-4, and PBA-W8-9). The interaction between the water molecules and the B(OH)$_{2}$ group occurs through the proton donor-acceptor for all the reported configurations. Generally, it has been noted that the involvement of B(OH)$_{2}$ in the hydrogen bond network generates a stable configuration. The stability of the structures of the microhydrated PBA-W8 is governed by the type of its bonding hydrogen network. similarity between the configuration of the PBA-W8 and those neutral water clusters [22, 37] is observed. In addition, the structures of PBA-W$_n$ located in this work exhibit similar hydrogen bond networks as those of PhOH-W$_n$ reported previously by Malloum and Conradie [7].

**Fig. 8:**
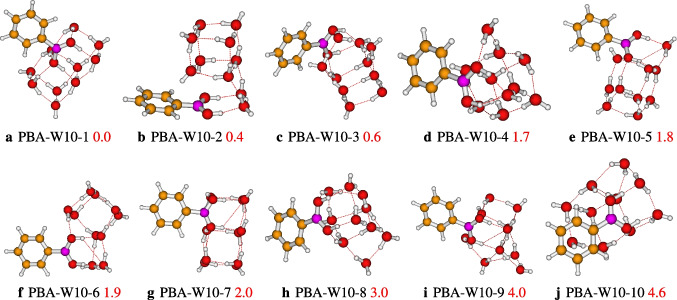
Different conformers of the phenylboronic acid in water for $n = 10$, optimized at the $\omega$B97X-D/def2-TZVP level of theory. The relative zero-point corrected energy of isomers in kcal/mol is represented by the numbers in red colorlabel

**Fig. 9:**
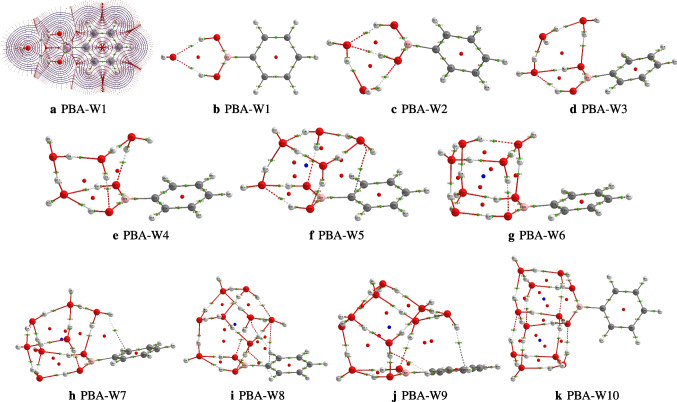
QTAIM analysis of the phenylboronic acid in water clusters, PBA(H$_2$O)$_n$, $n = 1-10$, using the most stable conformers: critical points and bond paths

We located ten isomers on the PES of the PBA-W$$_9$$, which are reported in Fig. 7. The most stable isomer, PBA-W9-1, has strong OH$$\cdots $$O and weak CH$$\cdots $$O hydrogen bonds. In addition to the two types of the hydrogen bonds, the QTAIM analysis of the PBA-W9-1 shows the presence of OH$$\cdots \pi $$ interaction (see Fig. 9). For all of the configurations, the interaction between solvent molecules(water) and the B(OH)$$_{2}$$ group (see Fig. 7) occurs through the proton donor-acceptor. The group B(OH)$$_{2} $$ and the solvent molecules(water) form a cyclic cubic-like configuration similar to neutral water clusters [22, 26, 35]. Regarding the phenylboronic acid solvated with ten explicit water molecules, we have reported in Fig. 8 ten stable structures, whose relative energy varies from 0.0 to 4.6 kcal/mol. According to the case of lower-sized clusters, in the case of decamer, it is noted that the most stable configuration is also the isomer where the solvent molecules(water) interact with a high number of strong and weak hydrogen bonds, respectively, the OH$$\cdots $$O bonds and the CH$$\cdots $$O bonds.

From the examination of the structures of PBA-W$_n$, from monomer to decamer, one can retain the following conclusions.

- The molecules of sovent(water) interact strongly with the B(OH)$_2$ group of PBA.
- The two hydroxyl functional groups in B(OH)$_2$ and the *n* water molecules reproduce the configurations of neutral water clusters of size $n+2$. Thus, the structures of PBA-W$_n$ are similar to those of W$_{n+2}$. Similarly, the structures of PBA-W$_n$ resemble those of PhOH-W$_{n-1}$.
- The most stable structures of PBA-W$_n$ are stabilized by strong bondings hydrogen, OH$\cdots$O and weak hydrogen bonds, CH$\cdots$O. Some isomers are further stabilized by OH$\cdots \pi$ hydrogen bonds.
- The phenyl group of PBA is rarely involved in the interaction with the water molecules. Therefore, the B(OH)$_2$ group is much more hydrophilic than the phenyl group.
- The stability of isomers of PBA-W$_n$ is governed by the number of OH$\cdots$O hydrogen bonds.

### QTAIM analysis of non-covalent bondings

Further details about the different bonding interactions bet-ween the phenylboronic acid and the solvent molecules(water) are provided using QTAIM analysis performed on the most stable isomers of the investigated clusters. For the located low-lying energy conformers in Fig. 9 are reported the different parameters as critical points and bond paths. Additional to the electron density ($\rho$), we have specifically, for the phenylboronic acid-water monomer, reported, also the atomic basins in the phenylboronic acid plane and the two-dimensional contour of $\rho$ (see Fig. 9). At all bond critical points (BCPs), the quantitative data are reported in the supporting material. The 2D contour electron density as well as the atomic basins of PBA-W$_1$ analysis presenting two OH$\cdots$O hydrogen bonds according to the bond paths and the critical points. These hydrogen bonds present the same values of $\rho$ and $\nabla ^2 \rho$ (the Laplacian of the electron density ) at the corresponding bond critical points. At the bond critical points, the positive values of $\nabla ^{2} \rho$ (0.0738 au) indicate a non-covalent bonding (attributed to the hydrogen bonds, OH$\cdots$O). In addition, for the two hydrogen bonds OH$\cdots$O, the value of the electron density is 0.0188 au, which indicates that the two hydrogen bonds have the same strength ($C_{2v}$ symmetry point group).

**Table 1:** Minimum and maximum values of $\rho$ and $\nabla ^{2} \rho$ of the most stable conformers of the phenylboronic acid-water clusters at bond critical points, for *n* = 1–10, at the $\omega$B97X-D/def2-TZVP level of theory

	$\rho (ea^{-5})$		$\nabla ^{2}(ea^{-5}) \rho$	
Bondig	Min	Max	Min	Max
OH$\cdots$O	0.0115	0.0548	0.0435	0.1296
CH$\cdots$O	0.0049	0.0051	0.0195	0.0232
OH$\cdots \pi$	0.0078	0.0088	0.0254	0.0279
O$\cdots$C	0.0057	0.0065	0.0185	0.0226

The interval defined the value of $\rho$ and $\nabla ^{2}\rho$ of the most stable isomers of the phenylboronic acid-water clusters at bond critical points have been reported in Table 1. It has been demonstrated that the strength of non-covalent bondings (especially hydrogen bonds) is proportional to the value of the electron density at bond critical point [38–45]. Thus, the higher value of $\rho$ at BCP corresponds to the stronger bond. Examination of the values of $\rho$ as reported in Table 1 shows that the OH$\cdots$O hydrogen bonds are the strongest non-covalent interactions in PBA-W$_n$. In addition, for the specific case of PBA-W$_n$ studied in this work, OH$\cdots$O type are much stronger hydrogen bonds than bonding hydrogen, CH$\cdots$O. In Fig. 9, we can observe that in the clusters of phenylboronic acid-water, the non-covalent bonds are dominated by the bonding hydrogen type OH$\cdots$O, established mainly between the water molecules. After analysis, we can see in (Fig. 9 and Table 1) two other non-covalent bondings have identified in the clusters of phenylboronic acid-water which are the OH$\cdots \pi$ bond and the O$\cdots$C bond, in additional to the hydrogen bonds, OH$\cdots$O and the hydrogen bonds CH$\cdots$O. We have also noted that the number OH$\cdots \pi$ and O$\cdots$C bonds identified in this work are few.

**Fig. 10:**
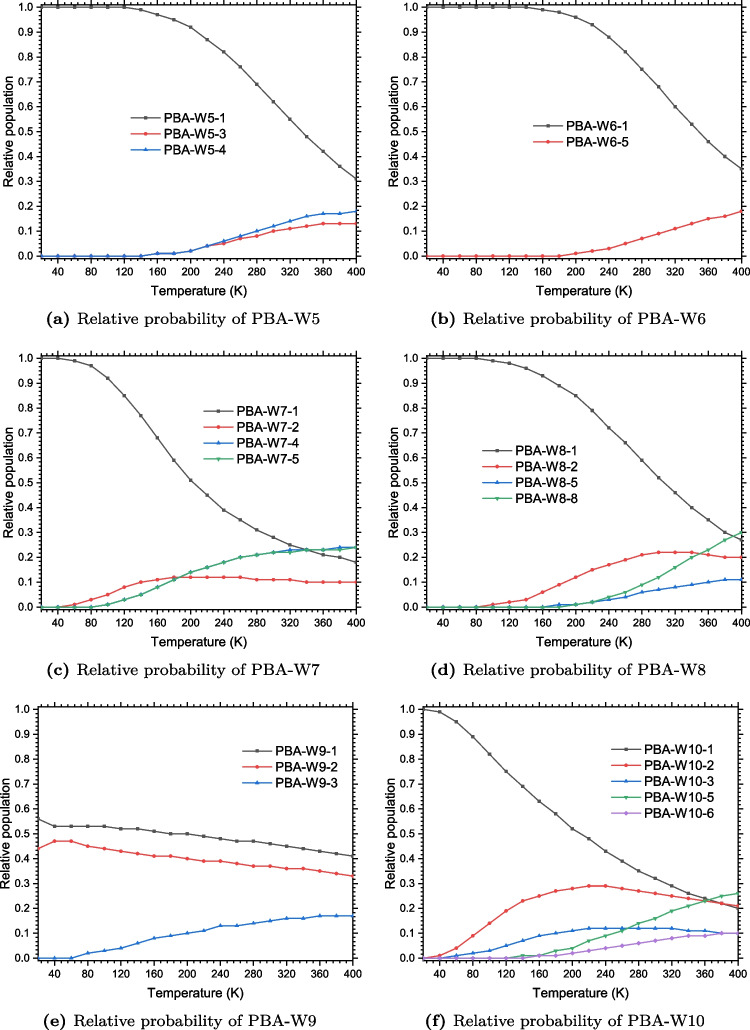
Relative population of the solvated phenylboronic acid, PBAW$_{5-10}$ with explicit water molecules, computed using Eq. 5

### NBO analysis

Natural bond orbital (NBO) analysis has been performed after QTAIM on the most stable configurations of phenylboronic acid on the sizes from $n=1$ to $n=10$ at SMD applied to $\omega$B97X-D/def2-TZVP level of theory. NBO analysis is an important tool for better understanding the electron density delocalization from the occupied Lewis-type (bond or lone pair) NBO orbitals, and formally unoccupied (antibonding and Rydgberg) non-Lewis-type NBO orbitals correspond to a stabilizing donor-acceptor interaction. The stabilization of orbital interaction is evaluated from the energy difference between the interacting orbitals. Therefore, the interaction with the strongest stabilization between effective donors and effective acceptors is determined from the second-order perturbation interaction energy E(2) in terms of NBO analysis. E(2) estimated from the Eq. 6

6 $$\begin{aligned} E(2)=\Delta E_ij=q_i \dfrac{F^2(ij)}{\varepsilon _j-\varepsilon _i} \end{aligned}$$

represents the estimate of the off-diagonal NBO Fock matrix element. where q$_i$ is the donor orbital occupancy, $\varepsilon _i$ and $\varepsilon _j$ are diagonal elements (orbital energies) and F(i,j) is the off-diagonal Fock matrix element. The obtained results reported in the supporting information show that for $n=1$ the strongest stabilization takes place between LP(2)O1$\mapsto$ BD*(1)O18-H19 with E(2)=7.27 kcal/mol; for $n=2$, the strongest stabilization is observed between LP(2)O1$\mapsto$ BD*(1)O4-H6 with E(2)=16.66 kcal/mol; for $n=3$, the interaction with strongest stabilization is the interaction between LP(2)O4$\mapsto$ BD*(1)O22-H23 with E(2)=16.69 kcal/mol;for $n=4$, we have the interaction between LP(2)O7$\mapsto$ BD*(1)O10-H11 with E(2)=21.22 kcal/mol; for $n=5$, we have the interaction between LP(2)O4$\mapsto$ BD*(1)O10-H11 with E(2)=18.99 kcal/mol which is shown the strongest stabilization for this size; for $n=6$, the strongest stabilization is indicate by the interaction between LP(2)O16$\mapsto$ LP*(1)H34 with E(2)=30.87 kcal/mol; for $n=7$, the strongest stabilization is between LP(2)O4$\mapsto$ LP*(1)H37 with E(2)=31.34 kcal/mol; for $n=8$, we have LP(2)O16$\mapsto$ BD*(1)O22-H23 with E(2)=23.39 kcal/mol; for $n=9$, we have LP(2)O19$\mapsto$ BD*(1)O25-H26 with E(2)=22.89 kcal/mol; for $n=10$, the strongest stabilization is observed between LP(2)O25$\mapsto$ LP*(1)H46 with E(2)=28.00 kcal/mol. BD* represents 2-center antibond type $\sigma *$; LP for 1-center valence lone pair; LP* for empty 1-center valence orbital or unfilled valence-shell nonbonding. For all the sizes, the strongest stabilization is dominated by the interaction between LP(2)O4$\mapsto$ LP*(1)H37, and all the Lewis-type or the donors are dominated by LP(2). Most unoccupied non-Lewis-type are BD*(1) type $\sigma *$, and in addition to this type, the type LP* is also observed. In the case of the occupied Lewis-type, the interactions are dominated by the interaction type LP. In additional, poor stabilization is also indicated by the interaction between LP ( 1) O 1$\mapsto$RY*( 3) C 16 with E(2)= 0.11 kcal/mol, BD ( 1) O 19 - H 20 $\mapsto$ BD* ( 2) C 26 - C 27 with E(2)= 0.12 kcal/mol and BD ( 2) C 32 - C 33 $\mapsto$ BD*( 1) O 19 - H 21 with E(2)=0.50 kcal/mol Where BD(2) and BD*(2) represent occupied and unoccupied lewis-type respectively type $\pi$ and $\pi *$. This poor stabilization is materialized by a low value of the E(2).

### Relative population of the hydrated phenylboronic acid

On the stability of the studied clusters, the effects of temperature are evaluated from Eq. 5, which represents the Boltzmann distribution. We have calculated the probabilities (relative population) of retained configurations of the phenylboronic acid-water clusters for the temperature values between 20 and 400 K. We have plotted the calculated probabilities of PBA-W$_n$ from $n=5$ to $n=10$ as a temperature function in (Fig. 10). In the supporting information, we reported the probabilities of lower-sized clusters (from $n=1$ to $n=4$). The results indicate that the most stable conformers dominate the population of these small-sized clusters. For the case of the tetramer, for which the three iso-energetically structures, the isomer PBA-W4-3 is the most dominant, while other configurations also contribute significantly to the population. The results of all the investigated clusters, PBA-W$_n$, at all temperatures, show that the most favored structures are the most stable(see Fig. 10). Nevertheless, at high temperatures, there is competition between other isomers, and these isomers contribute significantly to the population of the clusters. It is worth mentioning that the plots of Fig. 10 consider only isomers with a contribution of more than 10% to the population of the clusters.

### Conformers of neutral water clusters

**Fig. 11:**
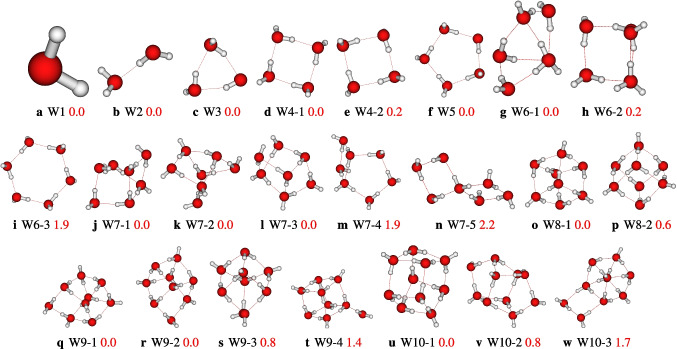
Geometries of neutral water clusters from monomer to decamer as optimized at the $\omega$B97X-D/def2-TZVP level of theory using the SMD implicit solvation model. The relative zero-point corrected energies of isomers in kcal/mol are represented by the numbers in red color. Relative energies are meaningless for cluster sizes with only one structure

In this work, the adopted solvation model of density (SMD) used to calculate the hydration free energy of the phenylboronic acid shows that the geometries of neutral water clusters are needed to compute these energies. In this work, we concentrated mainly on the structures that contribute significantly to the population of the clusters based on previous works [22, 26]. Thus, for the neutral water monomer, dimer, trimer, and pentamer, only one stable configuration for each size is considered (see Fig. 11). For the tetramer, two cyclic isomers with similar energies, W4-1 and W4-2, are considered. Regarding the water hexamer, three isomers are considered (see Fig. 11). These structures have been re-optimized in this work at the $\omega$B97X-D/def2-TZVP level of theory to compute the hydration free energies using the SMD implicit solvation model.

Regarding the structures of the water heptamer, five stable configurations are considered, and the located geometries are reported in Fig. 11 with relative energy varying from 0.0 to 2.2 kcal/mol. The observation of the results shows the three most stable geometries of the water heptamer, W7-1, W7-2, and W7-3, presenting the same relative energy(iso-energetically)(see Fig. 11). For the case of the octamer water clusters, two configurations are located as most stables, which are reported in Fig. 11. The two geometries have cubic-like configurations. For the water nonamer, four structures are considered in this work, while three isomers of the water decamer are considered as reported in Fig. 11. This work has re-optimized these structures at the $\omega$B97X-D/def2-TZVP level of theory to compute the hydration free energies using the SMD implicit solvation model.

### Hydration enthalpy and free energy

**Fig. 12:**
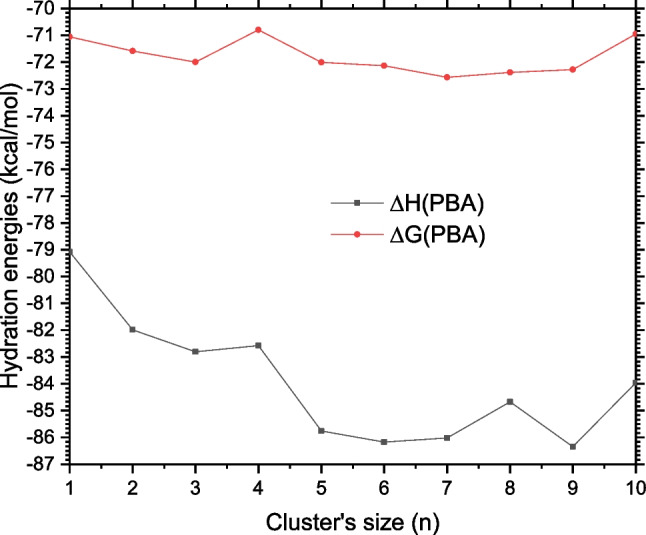
Free energy of hydration and enthalpy of hydration of the phenylboronic acid as a function of the clusters size *n* at atmospheric pressure and room temperature

The structures of PBA-W$_n$ and those of W$_n$ reported previously are used to estimate the free energy of hydration and the enthalpy of hydration of PBA. We used all the possible configurations for each cluster size as indicated in the “Rela-tive population of the hydrated phenylboronic acid” section, weighted by their relative probabilities. Before properly presenting phenylboronic acid’s determined free energies of hydration and enthalpies of hydration, we want to provide some information about these energies for the reader.The hydration free energies are measurable physical quantities used to understand some properties, such as reactivity and kinetics evolution of the solvated molecules. They are also used to study several biological and chemical processes, as indicated in the work previously reported by Malloum, Fifen, and Conradie [46].The free energy of hydration and the enthalpy of hydration of the phenylboronic acid in water variation are plotted in Fig. 12 as a cluster size *n* function at 298.15 kcal/mol defined as a room temperature and at atmospheric pressure. Examination of the curves in Fig. 12 shows that the free energy of hydration and the hydration enthalpy of PBA vary negligibly as the cluster’s size function. The changes are lower than $\sim$8 kcal/mol for both the enthalpy of hydration and the free energy of hydration. The trend of the curves indicates a convergence of the values from $n=5$. Therefore, to calculate PBA’s free energy of hydration and the hydration enthalpy at room temperature, we averaged over the values estimated for $n=5-10$. Finally, we estimated respectively the free energy of hydration and the enthalpy of hydration of the phenylboronic acid to $-$72.1 kcal/mol and $-$85.5 kcal/mol when the explicit solvents are added. In addition, without the explicit solvents, the values are respectively evaluated to $-$75.7 kcal/mol and $-$75.7 kcal/mol. It is clear that when the explicit solvation is not used, the hydration enthalpy and the hydration Gibbs free energy are estimated to be identical. This result highlights the importance of considering explicit solvation in the calculation of the hydration free energy and enthalpy of organic molecules.

**Fig. 13:**
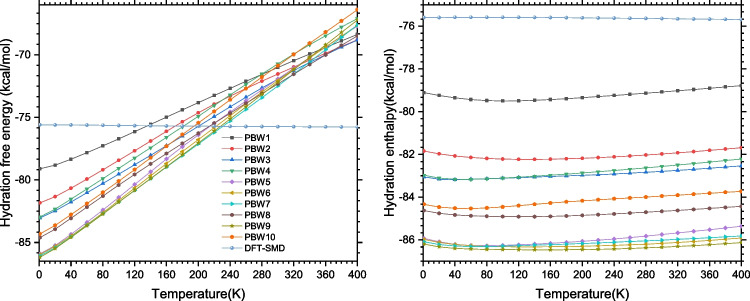
Free energy of hydration and enthalpy of hydration of phenylboronic acid using the values of the temperatures varying from 20 to 400 K

Ranaudo et al. [15] have reported the free energy of hydration of the anionic form of phenylboronic acid PhB(OH)$_{3}^{-1}$ using three different models based on DFT and molecular dynamics simulations. The evaluated hydrated free energies with these three models are $-$82.7 kcal/mol, $-$67.2 kcal/mol, and $-$67.6 kcal/mol [15]. It should be noted that no value of the solvation free energy of PhB(OH)$_{2}$ (PBA) has been reported previously in the literature. Nevertheless, the free energy of hydration of PhB(OH)$_{2}$ estimated in this work is of similar value to those of PhB(OH)$_{3}^{-1}$ estimated by Ranaudo et al. [15]. Despite the absence of reference experimental data on the free energy and enthalpy of hydration of PBA, the values estimated in this work can be considered reliable. It is important to note that the cluster continuum solvation model used in this work has been used previously to estimate the enthalpy of hydration of phenol in agreement with experiment [7].

Before studying the temperature effects on the free energy of solvation and the solvation enthalpy for the temperatures defined from 20 to 400 K, we first examined the effects of explicit solvation. In Fig. 13, we plotted as a temperature function the computed free energy of solvation and the enthalpy of solvation of phenylboronic acid for $n=1-10$, and these oscillations can be seen. As indicated in the results, the enthalpy of hydration for a given cluster size varies slowly with changing temperature values. The variation of the hydration enthalpy from 20 to 400 K is less than 1.0 kcal/mol. This variation shows that the enthalpy of hydration is temperature-independent. The temperature effects are unnecessary when considering the hydration enthalpy of the phenylboronic acid. Elsewhere, as shown in Fig. 13, the free energy of hydration varies almost linearly as a function of temperature. This linear variation indicates the importance of temperature’s effects on the hydration free energy of the phenylboronic acid. Depending on the cluster size, from 20 to 400 K, the change of the hydration free energy can reach up to 19.0 kcal/mol. This change is considerable and justifies the need to consider temperature effects in determining the free energy of hydration of PBA. The fact that the hydration enthalpy is temperature-independent, combined with the linear variation of the hydration free energy, shows that the free energy of hydration variation of PBA depends on the curves-slope (the entropy of hydration). Thus, we can affirm that the phenylboronic acid’s free energy of hydration variation is governed by entropy.

## Conclusions

We provide in this work, the free energy and enthalpy of solvation of phenylboronic acid in water for temperatures varying from 20 to 400 K. The free energy and enthalpy of hydration are determined using the cluster continuum solvation model (CCM). Initially, we generated the configurations of the phenylboronic acid with explicit water molecules (PhB(OH)$_2$-(H$_2$O)$_n$ (or PBA-W$_n$) for $n=1-10$). In addition to the configurations of PBA-W$_n$, the CCM requires the structures of neutral water clusters (W$_n$). Therefore, representative structures of W$_n$ are retrieved from previous works. The optimization of the structures of PBA-W$_n$ and those of W$_n$ has been performed using the $\omega$B97X-D/def2-TZVP level of theory. As the structures of PBA-W$_n$ are provided in this work for the first time, detailed insights regarding the relative energies of the conformers and their hydrogen bonds network are provided. The hydrogen bond network of PBA-W$_n$ is studied using the QTAIM analysis. Furthermore, the stability of the structures of PBA-W$_n$ as a function of temperature is assessed.

The results show similarities between the configurations of the phenylboronic acid-water clusters and those of neutral water clusters (without the phenyl group of PBA). However, compared to neutral water clusters, we noted that the configurations of phenylboronic acid-water clusters are folded. This is due to the interaction between the B(OH)$$_{2} $$ group and water molecules. It has been found that the water molecules surrounding the PBA interact mainly with the B(OH)$$_{2} $$ group, while the interaction of the phenyl group with the water molecules is scarce. The two hydroxyl groups in B(OH)$$_{2}$$, along with the *n* water molecules, reproduce the configuration of neutral water clusters of size $$n+2$$. QTAIM analysis is performed on the most stable structures of PBA-W$$_n$$. It comes out that the geometries are stabilized by weak CH$$\cdots $$O hydrogen bonds and strong OH$$\cdots $$O hydrogen bonds. In addition, we have also identified two other bonding interactions: OH$$\cdots \pi $$ and O$$\cdots $$C (with small occurrence). The hydrogen bond network of PBA-W$$_n$$ is predominantly constituted of OH$$\cdots $$O hydrogen bonds. Furthermore, NBO analysis on the most stable structures of phenylboronic acid is performed. Examination of the temperature effects on the structures of PBA-W$$_n$$ shows that the most stable conformers dominate the cluster’s population.

The results indicate that explicit solvation has negligible effects on phenylboronic acid’s free energy and enthalpy of solvation. However, an acceptable convergence is reached for the hydration enthalpy from $n=5$. At room temperature, the free energy and enthalpy of hydration of PBA are evaluated to be $-$72.1 kcal/mol and $-$85.5 kcal/mol, respectively. Examining temperature effects indicates that the enthalpy of hydration is temperature-independent, while the free energy of hydration of the phenylboronic acid is linearly varying as a function of the temperature.

## Supporting information

This supporting information contains the relative population curves of PBA-W$_n$, $n=1-4$, the Cartesian coordinates of the all the structures of PBA-W$_n$ reported in this work, the QTAIM and NBO analysis data of the most stable structures of PBA-W$_n$.

## Data Availability

We provide in the manuscript or in the supporting information the data used in this work.
